# Salmagundi

**Published:** 1887-08

**Authors:** 


### SALMAGUNDI.

EDITED BY E. G. BETTY, D.D.S.

Dr. J. R. Callahan, of Hillsboro, Ohio, is the happy father of a bright little girl, recently arrived, whom he styles "Baby Mine."

---

A Thirteen-year-old girl, living in Jersey City, suffered last week with toothache, and one side of her face became swollen. The other night the abscess broke, and she bled to death before assistance arrived.—*Med. and Surg. Reporter.*

---

According to the *Dental Advertser* for July, The Lake Erie Dental Association is after brother Harlan's scalp for publishing a "query" purporting to be from a correspondent at Meadville, Pa. For the life of us we don't see what all the racket is about.

---

"Stop smoking," said a doctor to an ailing patient the other day, "and it will lengthen your days." The patient stopped. The doctor's prediction was verified. The first day, the patient declares, was as long as his whole previous life; and he was inclined to favor the idea "a short life and a merry one."

An Expensive Set of False Teeth.—Dr. Evans, the American dentist in Paris, made a set of teeth for an English lady, the ivories being carefully chosen from the mouths of twenty Breton girls, who submitted to the extraction for a pecuniary compensation. Shortly after the set was delivered, the lady traveled to Mentone, and was aroused from her bed by the recent earthquake. She is now back in Paris with sunken-in lips, having forgotten her teeth in the escape from the shaking Italian hotel. A fresh lot of peasant girls with sound teeth are now wanted.—*Medical and Surgical Reporter.*

---

A Medical Opinion.—" Doctor, what do you think of this new treatment of consumption by injecting carbonic acid into the sternum ? "

" Well, it certainly distracts the mind of the patient for a while, and may do good by its moral effect by making him think of his latter end; he becomes, as it were, conscious of his rectum, as the schoolboy rendered *mens conscia recti.* He may even " bacilli " enough to cherish the hope that he is getting cured, and is taking a short cut to health over this *pons asinorum,* but I regret to say that I think he will find that the road will end as it began, in gas."—*Exchange.*

---

Ozonic Ether.—This is absolute ether containing thirty volumes of peroxide of hydrogen in solution. Dr. Richardson, Dr. Day, and others have used it successfully in diabetes, while the former says, " In whooping-cough it is highly beneficial, acting almost as a charm."

Used as a gargle in scarlet fever it has been found eminently useful.

| | | |
|---|---|---|
| ℞ | Ozonic ether | ʒ ij |
| | Glycerin | ℥ ss |
| | Water, q. s. ad | ℥ viii |

An ointment has been used with equal success, externally.

| | | |
|---|---|---|
| ℞ | Lard | ℥ iv |
| | Ozonic ether | ℥ iv |
| | Benzoic acid | grs. xl |
| | Otto roses | drops iv |

Ozonic ether is also a good disinfectant.—*Medical World.*

ERE the *Register* reaches its readers again the International Medical Congress will be in session at Washington. To this meeting the Dental profession of the United States has looked forward for a couple of years past, notwithstanding the diversity of opinion and the pseudo opposition of well-known individuals. All differences have been made up and in consequence every one looks forward to the largest meeting in point of numbers and greatest interest in all that the profession holds dear.

Let every one, who can possibly do so, be present, for this is an opportunity that rarely occurs to meet the bright-lights from the world over. No one will ever regret that he laid aside the business of every day life to indulge himself with a vacation and at the same time gather material to lay up for future use.

A week spent at Washington will be an ever pleasant and profitable experience to look back upon, when business gets dull and "The rain begins to darken the drear Novembers" of a life merging into the unknown hereafter.

---

ESSENTIAL PRODUCTS OF EUCALYPTUS.—Adrian writes in *Les Nouveaux Remedes* that the sale of eucalyptol, one of the hydrocarbons of eucalyptus, has considerably increased.

All species of eucalyptus, and there are one hundred and fifty varieties in Oceanica, yield an essence or oil, but not all oils contain eucalyptol. The oil obtained from *eucalyptus globulus* is mostly of uniform quality and excellence; it is the only one distilled in Europe.

In the southern part of France the leaves of *eucalyptus globulus* yields 0.40 to 0.70 per cent of oil. For one kilogram of oil it is necessary to distil one hundred and fifty to two hundred kilograms of leaves.

To obtain eucalyptol, commercial oil of eucalyptus is distilled in Henninger & Lebel's apparatus for fractional distillation. By careful manipulation a eucalyptol boiling from 174° to 175° C. is obtained which will amount to 50 per cent. of quantity put in still.

Generally 600 grams of eucalyptol are obtained from 1 kilogram of refined oil of *eucalyptus globulus.*

It must always be borne in mind that oil of eucalyptus is a complex product, of which some varieties will not yield eucalyptol, and even this product cannot with any certainty be considered a definite compound.—*National Druggist.*

